# *C*‐mannosylation promotes ADAMTS1 activation and secretion in human testicular germ cell tumor NEC8 cells

**DOI:** 10.1002/1873-3468.70133

**Published:** 2025-08-06

**Authors:** Takato Kobayashi, Takehiro Suzuki, Ryota Kawahara, Natsumi Harai, Naoshi Dohmae, Siro Simizu

**Affiliations:** ^1^ Department of Applied Chemistry Faculty of Science and Technology, Keio University Yokohama Japan; ^2^ Biomolecular Characterization Unit RIKEN Center for Sustainable Resource Science Wako Japan

**Keywords:** ADAMTS1, *C*‐mannosylation, protein modification, testicular germ cell tumor, vasculogenic mimicry

## Abstract

*C*‐mannosylation is a protein glycosylation that regulates the functions of target proteins. Although it has been reported that a disintegrin and metalloproteinase with thrombospondin motifs 1 (ADAMTS1), an important spermatogenesis factor, is *C*‐mannosylated, the roles of *C*‐mannosylation in ADAMTS1 in testicular cells are still unclear. In this study, we found that ADAMTS1 is *C*‐mannosylated at Trp^562^ and Trp^565^ in testis germ NEC8 cells. To determine the roles of *C*‐mannosylation in ADAMTS1, we established cells expressing a *C*‐mannosylation‐defective ADAMTS1, in which *C*‐mannosylated tryptophan residues were replaced with phenylalanine residues (ADAMTS1/2WF). Processing and secretion of ADAMTS1/2WF were both inhibited compared to those of wild‐type. Moreover, wild‐type ADAMTS1 degraded aggrecan, whereas ADAMTS1/2WF could not. These results indicate the impact of *C*‐mannosylation on ADAMTS1 function.

## Graphical abstract

*C*‐mannosylation is a unique form of protein glycosylation. In this study, we demonstrated that ADAMTS1 is *C*‐mannosylated at Trp^562^ and Trp^565^ in human testicular germ cell tumor NEC8 cells. We found that *C*‐mannosylation of ADAMTS1 is essential for its secretion, processing, enzymatic activity, and ability to promote vasculogenic mimicry. These findings highlight the critical role of *C*‐mannosylation in regulating ADAMTS1 function.

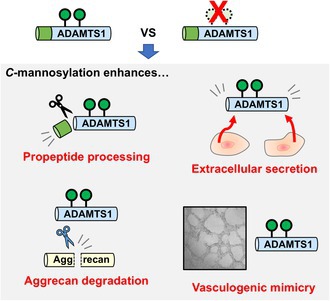

## Abbreviations

**ADAMTS1**, a disintegrin and metalloproteinase with thrombospondin motifs 1

**CBB**, Coomassie Brilliant Blue

**ECM**, extracellular matrix

**LC–MS**, liquid chromatography–mass spectrometry

**TGF‐β**, transforming growth factor‐β

**TSR1**, thrombospondin type‐1 repeat

**VM**, vasculogenic mimicry

ADAMTS1 was first cloned in 1997 by Matsushima and colleagues as an inflammation‐associated gene [1]. The ADAMTS family members consist of a signal peptide, propeptide, metalloproteinase domain, disintegrin, TSR1 domain, and spacer domain. Some ADAMTS members (ADAMTS2, 3, 10, and 17) promote polymerization of extracellular matrix (ECM) proteins, while other members catalyze the degradation of ECM proteins [2, 3]. ADAMTS1 is involved in organogenesis, vascular/lymphogenesis, and ovarian folliculogenesis by remodeling the ECM through degradation of substrates such as aggrecan and collagens [4, 5, 6, 7]. ADAMTS1 is also known to be associated with many diseases. The expression level of ADAMTS1 in the patients of oligozoospermia and azoospermia is lower than that of healthy control groups [8]. Furthermore, in this study, a negative correlation was observed between ADAMTS1 expression and sperm count and sperm motility, suggesting that ADAMTS1 might play an important role in spermatogenesis. Moreover, the expression level of ADAMTS1 is also known to be associated with tumor progression, and epigenetic modifications downregulate ADAMTS1 levels in many types of cancers, although a high expression level of ADAMTS1 is associated with cancer progression toward metastasis [9, 10, 11, 12, 13]. It has been reported that the expression level of ADAMTS1 is downregulated in pancreatic cancer patients; conversely, tumors with high ADAMTS1 expression are more likely to metastasize to lymph nodes [14]. Also, a previous study demonstrated that the knockout of ADAMTS1 inhibits vasculogenic mimicry (VM) formation, which is considered to promote cancer progression [15]. Although ADAMTS1 has been considered to be a positive regulator of tumor metastasis, the potential role in testicular cancer is not investigated.

Protein glycosylation is one of the most common posttranslational modifications occurring in the soluble proteins [16, 17, 18]. It is responsible for folding, solubility, stability, and protection from proteases. *C*‐mannosylation is a protein glycosylation in which mannose is attached to the N‐terminal tryptophan residue in the consensus sequence W‐X‐X‐W/C [19]. It has been reported that *C*‐mannosylation is required for protein folding, secretion, subcellular localization, intracellular signal transduction, and enzyme activity, like other protein glycosylation [20, 21, 22, 23, 24, 25]. ADAMTS1 is reported to be *C*‐mannosylated at Trp^562^, Trp^565^, and Trp^568^; however, the functional role of the *C*‐mannosylation in human testis cells is still obscure [26].

In this study, we elucidated that ADAMTS1 is *C*‐mannosylated at Trp^562^ and Trp^565^ in human testis germ tumor NEC8 cells, revealed by LC–MS/MS. These modifications affect its processing, secretion, VM formation, and enzymatic activity. Our data demonstrated a new insight into the roles of *C*‐mannosylation in ADAMTS1 and that *C*‐mannosylation might be a potential target for ADAMTS1‐related metastatic cancer.

## Materials and methods

### Cell culture

Human testicular germ cell tumor NEC8 cells (RRID:CVCL_1604, RIKEN BioResource Research Center, Tsukuba, Japan) were cultured in RPMI1640 medium (Nissui, Tokyo, Japan), which was supplemented with 10% FBS, 105 U·mL^−1^ penicillin G, 105 mg·L^−1^ kanamycin, 314 mg·L^−1^ L‐glutamine, and 2.25 g·L^−1^ NaHCO_3_, at 37 °C in a humidified incubator with 5% CO_2_. 293 T human embryonic kidney cells (RRID:CVCL_0063, RIKEN BioResource Research Center) were cultured in DMEM (Nissui, Tokyo, Japan) that was supplemented with 10% (v/v) FBS, 100 units·mL^−1^ penicillin G, 100 mg·L^−1^ kanamycin, 600 mg·L^−1^ L‐glutamine, and 2.25 g·L^−1^ NaHCO_3_ under 5% CO_2_ in a humidified incubator at 37 °C. All cell lines were maintained under mycoplasma‐free conditions and have been authenticated in the past 3 years.

### Construction of wild‐type ADAMTS1 and ADAMTS1/2WF overexpression plasmid

Human *ADAMTS1* was amplified with a cDNA library from the LNCaP human prostate cancer cell line. The sequences of primers that were used to amplify wild‐type full‐length *ADAMTS1* were as follows:

5′‐TTTTCTCGAGATGCAGCGAGCTGTGC‐3′ (forward1),

5′‐CCCCAAATGTAAACTGGCACTGCCGGTTGG‐3′ (reverse1),

5′‐AGTGCCAGTTTACATTTGGGGAGGACTCC‐3′ (forward2), and.

5′‐ GATGAGTTTTTGTTCACTGCATTCTGCCATTG‐3′ (reverse2).

These two DNA fragments were integrated by overlap extension PCR. To introduce a C‐terminal myc‐his_6_ tag, we performed a second PCR with primers that encode myc and his_6_ (designated as MH). The sequences of the tags were as follows: myc, 5′‐GAACAAAAACTCATCTCAGAAGAGGATCTG‐3′, and his_6_, 5′‐CATCATCACCATCACCAT‐3′. The obtained PCR product including ADAMTS1, myc, and his_6_‐encoding codon was subcloned into *XhoI*/*NotI* sites of CSII‐CMV‐IRES2‐Bsd (RIKEN BioResource Research Center). We simulated the folding status of ADAMTS1 wild‐type and its Trp562Phe and Trp565Phe variants using alphafold 3 (Fig. S1). The predicted structures of these proteins were highly similar. Therefore, we replaced the Trp562 and Trp565 residues in ADAMTS1 with phenylalanine (designated as ADAMTS1/2WF) by site‐directed mutagenesis using inverse PCR. The sequences of primers that were used for mutagenesis were as follows:

5′‐ATGTTCGGGCCTTGGGGAGACTGTTCGAGAAC‐3′ (forward),

5′‐TCCGAAGCTTCCATGAAAAGGCGTATCAAAATGC‐3′ (reverse). All plasmids have been analyzed by Macrogen Japan Corp. (Tokyo, Japan) to confirm that the DNA sequences are correct.

### Structural comparison

The amino acid sequence of human ADAMTS1 was obtained from the UniProt database (Q9UHI8; ATS1_HUMAN) and the folding status was simulated by alphafold 3 [27]. Because the mature form of ADAMTS1 is processed, the N‐terminal signal peptide and propeptide were removed, and then the simulation was performed. We replaced the Trp562 and Trp565 residues in ADAMTS1 with phenylalanine residues to simulate the conformation of ADAMTS1/2WF.

### Establishment of wild‐type ADAMTS1 and ADAMTS1/2WF overexpression NEC8 cell lines

293 T cells were transfected with wild‐type ADAMTS1‐MH or ADAMTS1/2WF‐MH overexpression plasmid using Lentivirus High Titer Packaging Mix (Takara Bio Inc., Shiga, Japan) and cultured for 6 h. The cells were washed with phosphate‐buffered saline (PBS), which was changed to fresh medium for 24 h, and the lentivirus‐containing conditioned media was collected. NEC8 cells were infected with the conditioned media to promote ADAMTS1‐MH expression, and the cells were selected with 10 μg·mL^−1^ blasticidin S (FUJIFILM Wako Pure Chemical Corporation, Osaka, Japan) at 24 h after the infection. These selected cells were designated NEC8‐ADAMTS1‐MH cells and NEC8‐ADAMTS1/2WF‐MH cells.

### Purification of recombinant ADAMTS1‐MH

NEC8‐ADAMTS1‐MH cells were washed twice with PBS and cultured in serum‐free RPMI 1640 containing 75 μg·mL^−1^ heparin for 24 h. Heparin was used to release ADAMTS1‐MH from cell surface heparan sulfate proteoglycan. The conditioned media were collected and concentrated using an Amicon Ultra‐15 mL filter (Merck KGaA, Darmstadt, Germany). Subsequently, the sample was mixed with Ni‐NTA agarose beads for 2 h at 4 °C. The Ni‐NTA‐bound proteins were eluted with the elution buffer [300 mm NaCl, 2.7 mm KCl, 10 mm Na_2_HPO_4_, 1.8 mm KH_2_PO_4_, and 250 mm imidazole], and the eluted samples were electrophoresed on an SDS‐polyacrylamide gel. The gel was visualized with CBB staining.

### LC–MS/MS

Purified ADAMTS1‐MH was analyzed by SDS‐PSGE. After CBB staining, the gel pieces containing the visible band were reduced with dithiothreitol (DTT) and alkylated with acrylamide. The gel bands were digested with trypsin (TPCK‐treated; Worthington Biochemical, Lakewood, NJ) at 37 °C for 12 h. The resulting peptides were analyzed by LC–MS/MS using a Vanquish Neo UHPLC system coupled to an Orbitrap Exploris 240 mass spectrometer (Thermo Fisher Scientific, Waltham, MA). Peptide separation was performed on a nano‐ESI spray column (NTCC‐360; 0.075 mm internal diameter × 150 mm length, 3 μm, Nikkyo Technos Co., Tokyo, Japan) at a flow rate of 200 nL·min^−1^. The mobile phases consisted of 0.1% formic acid in water (solvent A) and 80% acetonitrile with 0.1% formic acid (solvent B). A linear gradient was applied, increasing solvent B from 0% to 40% over 20 min. LC–MS/MS data were acquired in Top 20 data‐dependent acquisition (DDA) mode. The acquired data were analyzed using proteome discoverer 3.1, integrated with Mascot server 2.8. Database searches were conducted with the following parameters: enzyme, trypsin; fixed modification, none; variable modifications, acetyl (protein N‐term), Gln → pyro‐Glu (N‐term Q), oxidation (M), propionamide (C), Hex (W); monoisotopic mass values; peptide mass tolerance, ±15 ppm; fragment mass tolerance, ±30 mmu; maximum missed cleavages, 3; and instrument type, ESI‐TRAP. MS chromatograms of unmodified and mono‐, di‐, and tri‐hexosylated peptides were generated using qual browser 4.1.31.9 (Thermo Fisher Scientific).

### Western blot

Cells were lysed in lysis buffer [50 mm Tris–HCl (pH 7.5), 150 mm NaCl, 0.1% (w/v) SDS, 1% (v/v) Triton X‐100, 1% (w/v) sodium deoxycholate, and 1 mm phenylmethylsulfonyl fluoride] with sonication, and the lysates were centrifuged at 20 000× ***g*** for 10 min. The amounts of proteins were measured by protein assay dye reagent (Bio‐Rad Laboratories, Inc., Hercules, CA). Loading buffer [350 mm Tris–HCl (pH 6.8), 30% (w/v) glycerol, 0.012% (w/v) bromophenol blue, 6% (w/v) SDS, and 30% (v/v) 2‐mercaptoethanol] was added to each lysate, and the lysates were boiled for 3 min at 98 °C. The boiled lysates were loaded onto SDS‐polyacrylamide gels, transferred to PDVF membranes, and immunoblotted with anti‐c‐myc (purified from 9E10 cells of the Developmental Studies Hybridoma Bank), anti‐α‐tubulin (T5168; Merk KGaA) anti‐GST (sc‐138; Santa Cruz Biotechnology, Inc., Dallas, TX), anti‐p‐Akt (S473) (4060; Cell Signaling Technology, Danvers, MA), anti‐Akt (9272; Cell Signaling Technology), anti‐p‐ERK1/2 (T202/Y204) (9101; Cell Signaling Technology), and anti‐ERK1/2 (sc‐514302, Santa Cruz Biotechnology, Inc.) antibodies. Signals were detected with Immobilon Western Chemiluminescent HRP substrate (Merk KGaA).

### Semiquantitative RT‐PCR

Total RNAs were extracted from cultured cells with RNA extraction buffer [38% (w/w) phenol, 0.8 M guanidine thiocyanate, 0.4 M ammonium thiocyanate, 0.1 M sodium acetate, and 0.5% (v/v) glycerol], and 2 μg of the total RNAs were used for the reverse‐transcription reaction with the High‐Capacity cDNA reverse‐transcription kit (Thermo Fisher Scientific, Inc.). The obtained cDNA was used for PCR amplification. The sequences of primers that were used to perform the PCR amplification were as follows:

Exogenous ADAMTS1‐MH forward, 5′‐AGTGCCAGTTTACATTTGGGGAGGACTCC‐3′.

Exogenous ADAMTS1‐MH reverse, 5′‐AGATGAGTTTTTGTTCTGGACAGTCCTCAAG‐3′.

GAPDH forward, 5′‐GATTCCACCCATGGCAAATTCC‐3′.

GAPDH reverse, 5′‐CACGTTGGCAGTGGGGAC‐3′.

### Evaluation of ADAMTS1 secretion level

Cells were washed twice with PBS and cultured in serum‐free RPMI 1640 medium with 75 μg·mL^−1^ heparin for 24 h. The conditioned media were mixed with Ni‐NTA agarose beads for 2 h at 4 °C, and the Ni‐NTA‐bound proteins were eluted with elution buffer. The amounts of proteins in the conditioned media were normalized to the concentration of proteins from the cell lysates. The loading buffer was added to the eluted samples, and the mixtures were boiled for 3 min. The proteins were separated by SDS‐PAGE and analyzed by western blot with anti‐c‐myc or anti‐α‐tubulin antibodies.

### Evaluation of ADAMTS1 enzymatic activity

Cells were washed twice with PBS and cultured in serum‐free RPMI 1640 medium with 75 μg·mL^−1^ heparin for 24 h. The conditioned media were collected and concentrated using an Amicon Ultra‐15 mL filter. Subsequently, the sample was mixed with Ni‐NTA agarose beads for 2 h at 4 °C. The Ni‐NTA‐bound proteins were eluted with the elution buffer and measured for concentration. Glutathione *S*‐transferase (GST)‐tagged aggrecan of the interglobular domain was purified from *E*. *coli* as described before [28]. The GST‐tagged aggrecan and recombinant ADAMTS1 or ADAMTS1/2WF were incubated for 24 h at 37 °C in reaction buffer [50 mm Tris–HCl (pH 6.8), 150 mm NaCl, and 5 mm CaCl_2_]. Then, the solution was mixed with Glutathione Sepharose 4B (Cytiva, Marlborough, MA) for 2 h at 4 °C. The Glutathione Sepharose 4B‐bound proteins were eluted by boiling for 5 min. The proteins were separated by SDS‐PAGE and analyzed by western blotting with an anti‐GST antibody.

### VM assay

The in vitro VM assay was conducted as described before [29, 30, 31]. First, 96‐well plates were coated with 40 μL Matrigel^®^ Growth Factor Reduced (Corning, Corning, NY) and incubated at 37 °C for 30 min. The cells were suspended in culture medium and seeded at 4.8 × 10^4^ cells/well into a Matrigel^®^‐coated 96‐well plate. The cells were incubated at 37 °C for 24 h. In each well, four independent, randomly selected fields were captured by phase‐contrast microscopy (Leica DMi1; Leica), and the number of tubes that consisted of cells was counted.

## Results

### Human ADAMTS1 is *C*‐mannosylated at Trp^562^ and Trp^565^ in NEC8 cells

**Fig. 1:**
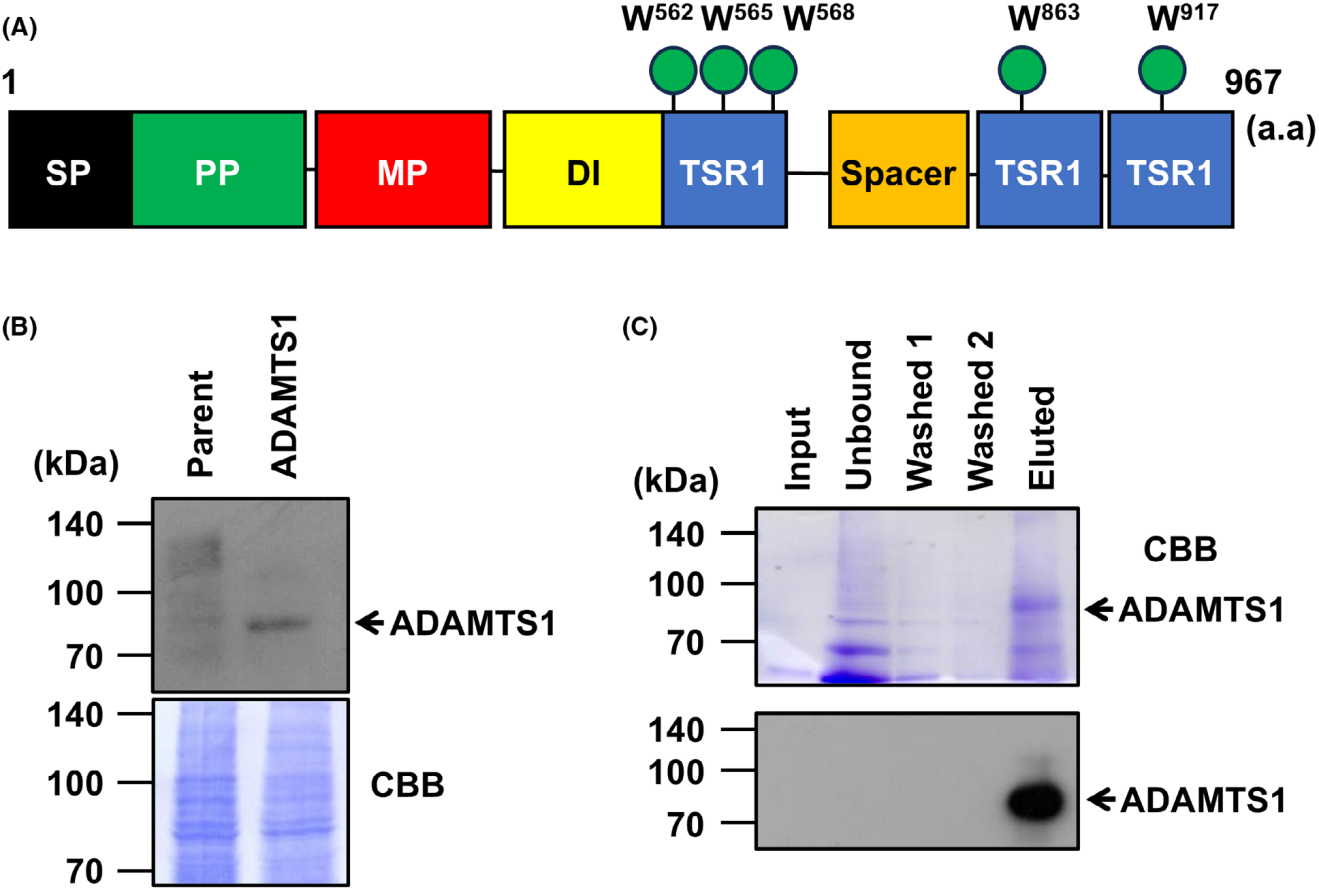
Recombinant ADAMTS1 was purified for mass spectrometry. (A) Schematic of human a disintegrin and metalloproteinase with thrombospondin motifs 1 (ADAMTS1) primary structure. The black box, the green box, the red box, the yellow box, 3 blue boxes, the orange box, and 5 green circles represent signal peptide (SP; 1–49 a.a.), propeptide (PP; 50–252 a.a.), metalloprotease (MP; 258–467 a.a.), disintegrin (DI; 476–559 a.a.), thrombospondin type‐1 repeat (TSR1; 559–614, 854–905, and 908–967 a.a.), spacer (725–849 a.a.), and putative *C*‐mannosylation sites (562, 565, 568, 863, and 917 a.a.), respectively. (B) Establishment of an ADAMTS1‐overexpressing cell line. Parental and NEC8‐ADAMTS1‐myc‐his_6_ (MH) cells were cultured in RPMI1640 medium. Total cell lysates were collected and electrophoresed on an SDS‐polyacrylamide gel and immunoblotted with an anti‐c‐myc antibody. Polyvinylidene fluoride (PVDF) membrane was stained with Coomassie Brilliant Blue (CBB) solution and used as a loading control. (C) Purification of recombinant ADAMTS1 protein. NEC8‐ADAMTS1‐MH cells were cultured in serum‐free RPMI1640 medium with 75 μg·mL^−1^ heparin for 24 h. Conditioned media was collected, and secreted ADAMTS1 was concentrated with Ni‐NTA agarose. Samples were electrophoresed on an SDS‐polyacrylamide gel. The gel was visualized with CBB staining.

Human ADAMTS1 has 5 putative *C*‐mannosylation sites in the TSR1 domains (Fig. 1A). To examine whether these tryptophan residues are *C*‐mannosylated, we established C‐terminally myc‐ and his_6_‐tagged ADAMTS1‐overexpressing NEC8 cell lines, termed NEC8‐ADAMTS1‐MH cells (Fig. 1B). Although full‐length ADAMTS1 was 105 kDa, the band detected in the cell lysate and the conditioned medium was around 85 kDa. This band might be an active form of ADAMTS1 in which the propeptide was removed. To confirm whether ADAMTS1 is *C*‐mannosylated using LC–MS/MS, we purified recombinant ADAMTS1 from the conditioned medium of NEC8‐ADAMTS1‐MH cells with Ni‐NTA beads (Fig. 1C).

**Fig. 2:**
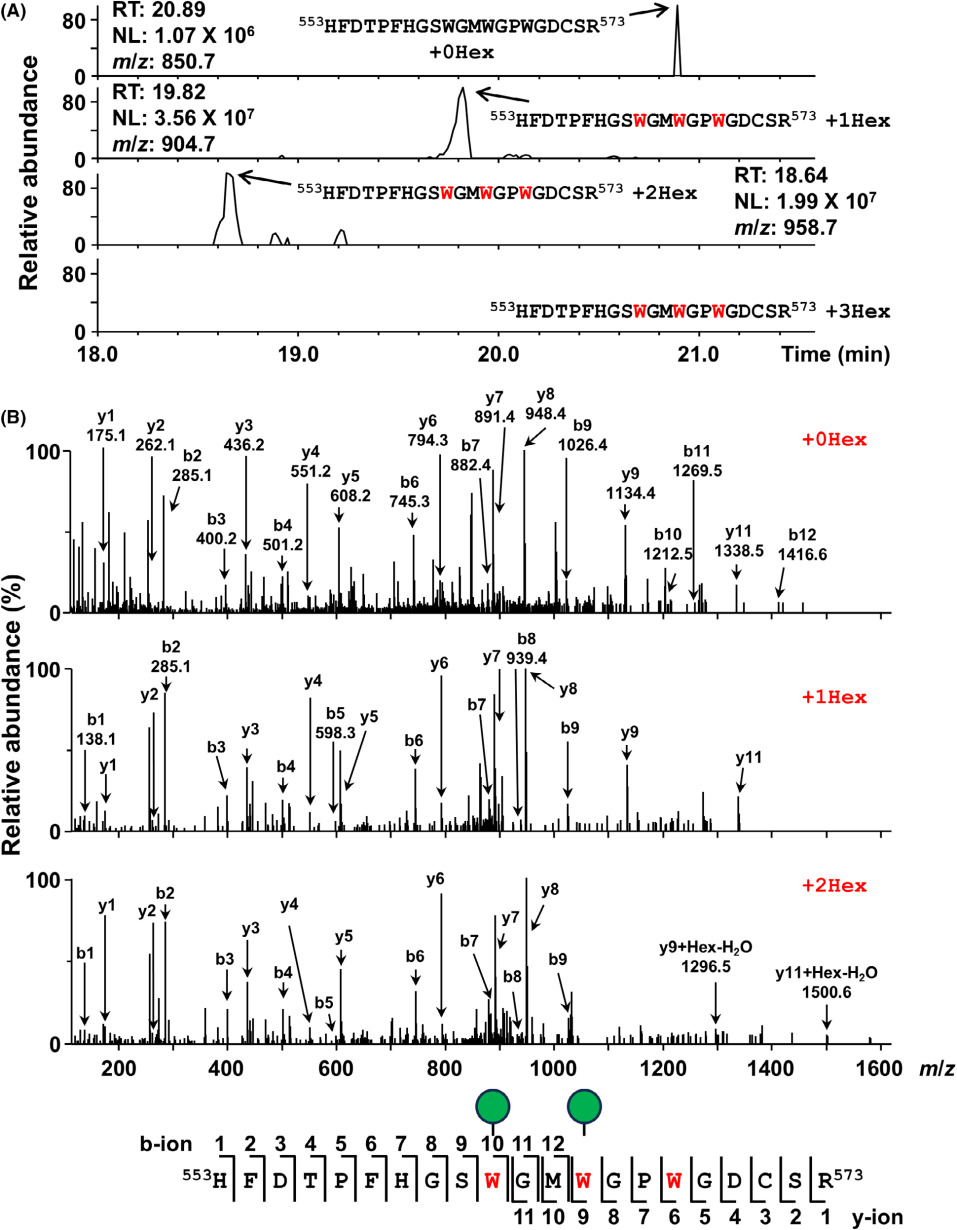
ADAMTS1 is *C*‐mannosylated at Trp^562^ and Trp^565^ in NEC8 cells. (A) The obtained samples were digested with trypsin or Asp‐N. The resulting peptides were analyzed by liquid chromatography–mass spectrometry (LC–MS)/MS. The ions of unmodified, mono‐mannosylated (+1Hex), and di‐mannosylated (+2Hex) H^553^‐R^573^ peptides were detected in the chromatogram. (B) The peptides were further analyzed by LC–MS/MS. Indicated b‐ions and y‐ions were detected, and Trp^562^ and Trp^565^ of ADAMTS1 were *C*‐mannosylated. Red texts indicate putative *C*‐mannosylation sites.

The purified sample was digested with trypsin, and the subsequent mixture of peptides was subjected to LC–MS. We detected ADAMTS1‐derived peptides lacking the signal peptide and propeptide regions (data not shown). In the ^553^HFDTPFHGSWGMWGPWGDCSR^573^ peptide, LC–MS analyses indicated 3 type peaks: unmannosylated (*m*/*z* = 850.7, RT = 20.89 min), mono‐mannosylated (*m*/*z* = 904.7, RT = 19.82 min), and di‐mannosylated (*m*/*z* = 958.7, RT = 18.64 min) (Fig. 2A). To identify which tryptophan residues are *C*‐mannosylated, we performed LC–MS/MS (Fig. 2B). Regarding the mono‐mannosylated peptide, the b1‐b9 and y1‐y9 ion peaks were not mannosylated, suggesting that the Trp^562^ was *C*‐mannosylated. Concerning the di‐mannosylated peptide, the y1‐y8 ion peaks were unmodified, but y9 ion peaks were observed +142 *m*/*z* (+1Hex‐H_2_O) compared to the y9 ion peak of the unmannosylated peptide. Furthermore, since the b1‐b9 ion peaks were not modified and the y11 ion was only increased +142 *m*/*z*, it was suggested that the remaining Trp^565^ was also *C*‐mannosylated. Therefore, our data indicated that Trp^562^ and Trp^565^ in the first TSR1 domain are *C*‐mannosylated in NEC8 cells—but not Trp^568^ or other Trp residues located in different TSR1 domains, such as Trp^863^ and Trp^917^.

### *C*‐mannosylation of ADAMTS1 regulates its processing and secretion

**Fig. 3:**
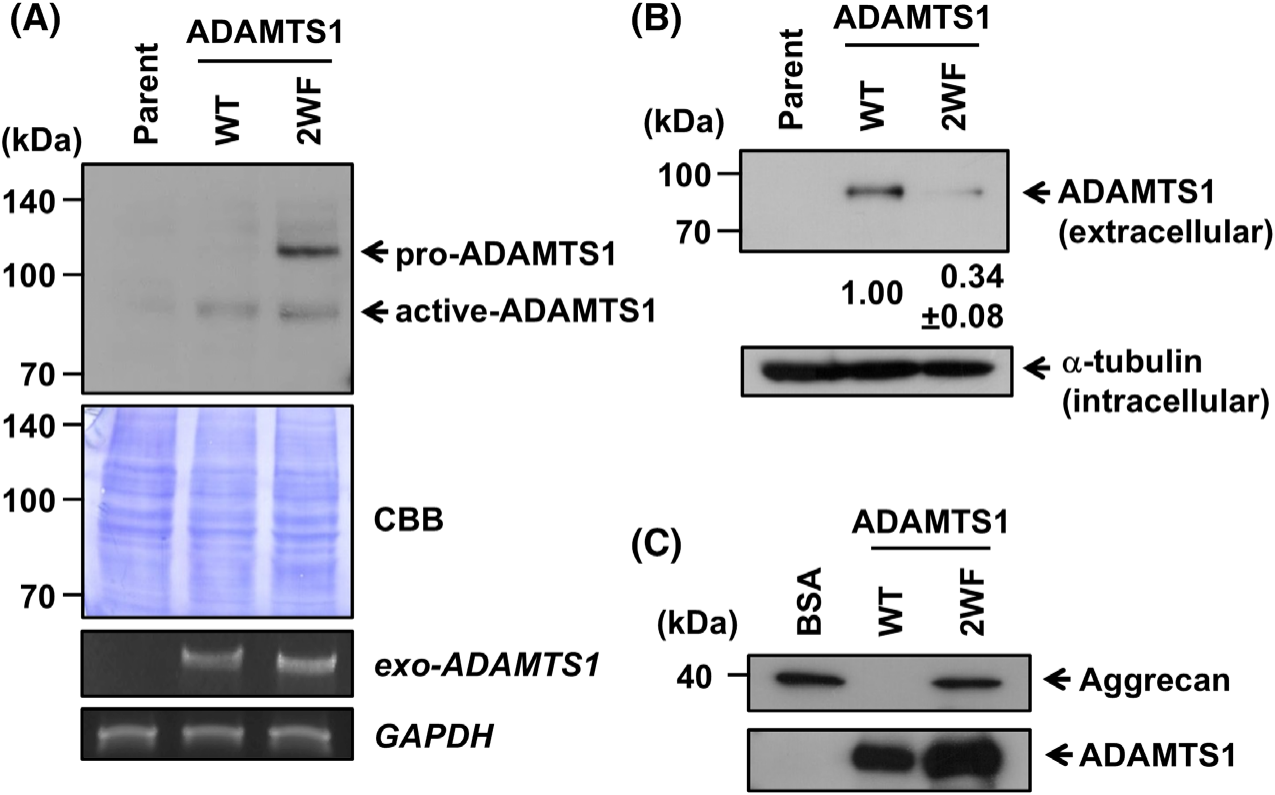
*C*‐mannosylation of ADAMTS1 regulates its processing and intracellular trafficking. (A) NEC8‐ADAMTS1/2WF‐MH cells were established. Parental NEC8 , NEC8‐ADAMTS1‐MH (WT), and NEC8‐ADAMTS1/2WF‐MH (2WF) cells were cultured in RPMI1640 medium. Total cell lysates were collected, electrophoresed on an SDS‐polyacrylamide gel, and immunoblotted with an anti‐c‐myc antibody. The total RNAs were isolated from each cell, and semiquantitaive RT‐PCR was performed. The indicated genes were amplified by semi‐quantitative PCR, and the resultant amplicons were electrophoresed on a 1% agarose gel with 0.5 μg·mL^−1^ ethidium bromide and observed under the LED. (B) Each cell line was cultured in serum‐free RPMI1640 medium with 75 μg·mL^−1^ heparin for 24 h. Conditioned media and total cell lysates were collected, and secreted ADAMTS1 was concentrated with Ni‐NTA agarose. Samples were electrophoresed on SDS‐polyacrylamide gels and immunoblotted with anti‐c‐myc and anti‐α‐tubulin antibodies. The signal intensities of secreted ADAMTS1‐MH and ADAMTS1/2WF‐MH were quantified and normalized to α‐tubulin expression using imagej software. (C) Recombinant ADAMTS1‐MH and ADAMTS1/2WF‐MH were incubated with GST‐tagged aggrecan for 24 h at 37 °C. The solution was mixed with Glutathione Sepharose 4B for 2 h at 4 °C. The samples were electrophoresed on SDS‐polyacrylamide gels and immunoblotted with anti‐GST and anti‐c‐myc antibodies. These experiments were repeated at least three times to confirm the experimental reproducibility.

*C*‐mannosylation regulates the subcellular localization and secretion of proteins [20, 21, 22, 23]. To determine the functional role of *C*‐mannosylation in ADAMTS1, we established the *C*‐mannosylation‐defective mutant‐overexpressing NEC8 cell line, termed NEC8‐ADAMTS1/2WF‐MH cells. In this cell line, both Trp^562^ and Trp^565^ are replaced with phenylalanine, and the expression was confirmed by western blot and semiquantitative RT‐PCR (Fig. 3A). Even though the mRNA level of ADAMTS1/2WF is comparable with wild‐type, the protein could be observed in both pro‐ and active form, whereas wild‐type ADAMTS1 could be observed only in active form (Fig. 3A), suggesting that *C*‐mannosylation is required for ADAMTS1 processing. To evaluate the effect of *C*‐mannosylation on the secretion of ADAMTS1, we detected secreted ADAMTS1 protein. As a result, the secretion level of ADAMTS1/2WF was decreased compared with wild‐type ADAMTS1 (Fig. 3B). Given the secretion level among these proteins, ADAMTS1/2WF is more likely to accumulate within the cell, as shown in Fig. 3A. These results indicate that *C*‐mannosylation of ADAMTS1 is necessary for its secretion. ADAMTS1 mediates cancer metastasis through degrading ECM components. Therefore, we examined the effect of *C*‐mannosylation on the ECM degradation activity of ADAMTS1. To assess the activity of ADAMTS1, we used aggrecan as a substrate of ADAMTS1 [32]. Recombinant wild‐type ADAMTS1 degraded aggrecan and completely diminished the signal of the original, but ADAMTS1/2WF did not show the activity, as the signal was comparable with the BSA‐treated band. The result suggests that *C*‐mannosylation of ADAMTS1 affects its ECM degradation activity.

### *C*‐mannosylation of ADAMTS1 influences vasculogenic mimicry and enzymatic activity

**Fig. 4:**
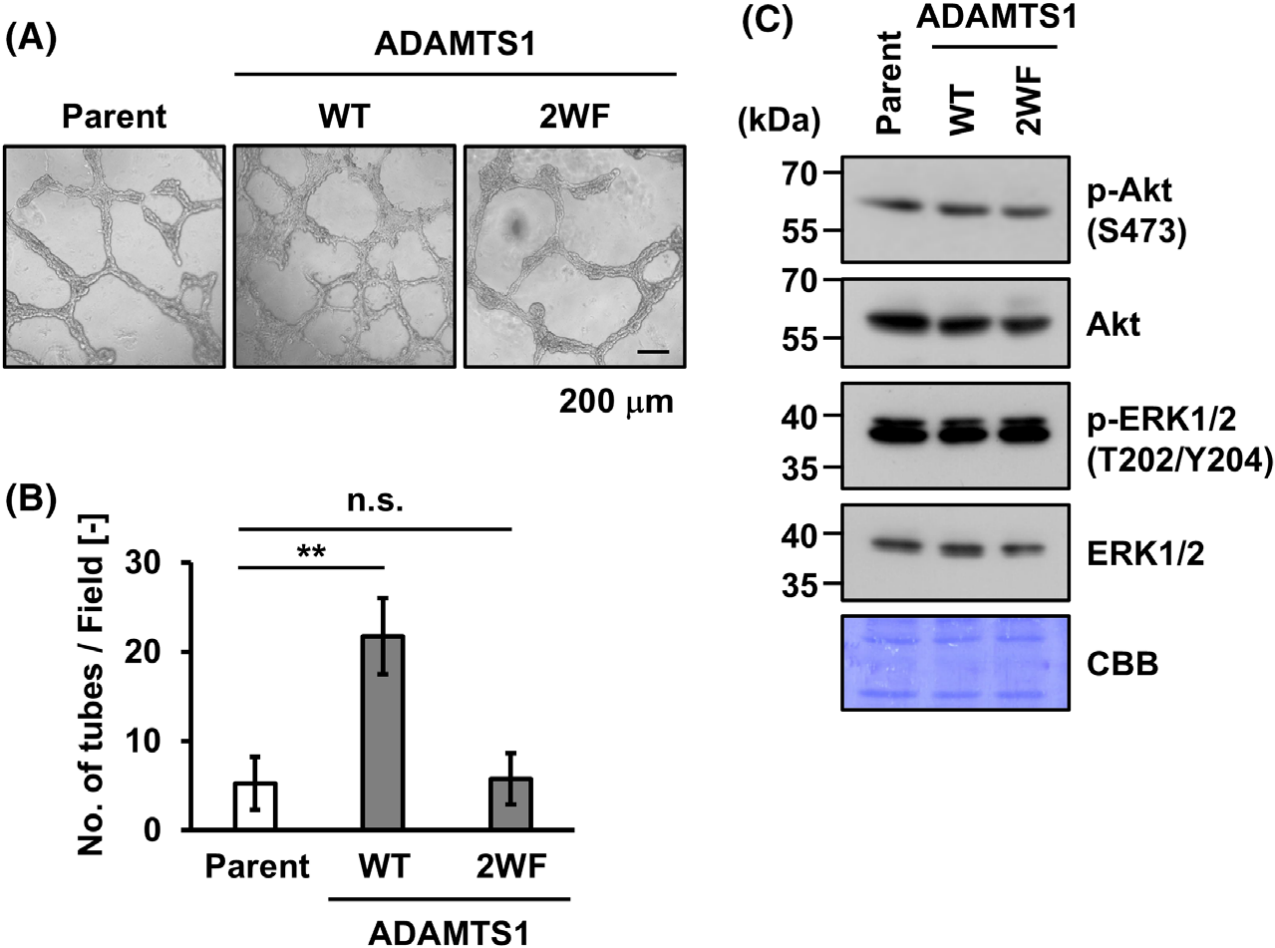
*C*‐mannosylation of ADAMTS1 influences vasculogenic mimicry formation. (A) Parental NEC8, NEC8‐ADAMTS1‐MH, and NEC8‐ADAMTS1/2WF‐MH cells were seeded on Matrigel‐coated wells (4.8 × 10^4^ cells/well). These photographs were taken at 24 h after seeding. (B) The number of tubes was counted in 4 randomly selected fields, and the statistical significance was analyzed by one‐way ANOVA with Tukey's test. (C) Cells were cultured in RPMI1640 medium. Total cell lysates were collected, electrophoresed on SDS‐polyacrylamide gels, and immunoblotted with anti‐p‐Akt (S473), anti‐Akt, anti‐p‐ERK1/2 (T202/Y204), and anti‐ERK1/2 antibodies. Scale bar, 200 μm. Data shown are the means ± SD. *P*‐value < 0.01 is indicated by **. n.s.; not significant. These experiments were repeated at least three times to confirm the experimental reproducibility.

ADAMTS1 has been implicated in the malignant transformation of cancer, including metastasis [9, 10, 11, 12, 13]. To uncover the effect of ADAMTS1 *C*‐mannosylation on cancer progression, we evaluated VM formation, which is closely correlated with cancer progression [33]. In a previous study, the knockout of the ADAMTS1 gene decreased the ability of VM [15], suggesting ADAMTS1 as a positive regulator for VM. Thus, we examined the effect of *C*‐mannosylation on ADAMTS1‐mediated VM formation using NEC8 cells. As shown in Fig. 4A,B, the overexpression of wild‐type ADAMTS1 increased VM formation compared with parental NEC8 cells; however, ADAMTS1/2WF did not promote VM formation. As shown in Fig. 3A, pro‐ADAMTS1/2WF accumulates in cells, so we examined the possibility of inhibitory effects of this protein on VM. Previous reports demonstrated that PI3K/Akt and MAPK signaling promote VM formation [34, 35] As a result, this signaling was not affected by ADAMTS1‐2WF (Fig. 4C). These data indicate that *C*‐mannosylation of ADAMTS1 positively influences VM formation in NEC8 cells.

## Discussion

*C*‐mannosylated proteins are broadly classified into two groups: proteins containing TSR1 domain(s) and the type I cytokine receptor superfamily. *C*‐mannosylation in the TSR1 domain is known to regulate its intracellular localization and secretion. For example, ADAMTSL1 is *C*‐mannosylated at Trp^42^ in the TSR1 domain [36]. A mutation in Trp^42^ to Arg in ADAMTSL1 has been identified in glaucoma patients, and this variant did not facilitate its secretion [37].

In the present study, we showed that Trp^562^ and Trp^565^ within the first TSR1 domain of ADAMTS1 are *C*‐mannosylated in NEC8 cells. *C*‐mannosylation in ADAMTS1, like other substrate proteins that contain TSR1 domains, also affected secretion. Shcherbakova *et al*. [38] showed that *C*‐mannosylation regulates stability and folding by modulating the flexibility of the Trp‐Arg ladder in the TSR1 domain. Thus, it is suggested that *C*‐mannosylation might stabilize ADAMTS1 conformation for secretion. We also found that *C*‐mannosylation positively regulates ADAMTS1 processing. ADAMTS1 is processed by a furin endopeptidase [39], which usually works in the Golgi apparatus [40]. Therefore, we speculated that a defect of *C*‐mannosylation in ADAMTS1 inhibits transport from the ER to the Golgi apparatus, resulting in the accumulation of pro‐form ADAMTS1.

In addition, *C*‐mannosylation is known to regulate other glycosylations in the same protein [22]. In the present study, our MS data revealed Thr^574^ in the N‐terminal TSR1 domain and Ser^869^ in the central TSR1 domain of ADAMTS1 were also *O*‐fucosylated (data not shown). A study suggested that *O*‐fucosylation occurs exclusively with *C*‐mannosylation [41]; thus, the role should be elucidated in a future study.

Moreover, we demonstrated that *C*‐mannosylation is required for ADAMTS1 enzymatic activity and VM formation (Figs 3 and 4). Regarding the enzymatic activity of ADAMTS1, it is known that the spacer domain and TSR1 domain may play important roles for binding with ECM components. On the other hand, only the N‐terminal TSR1 domain is not sufficient to recognize ECM [32]. In this study, we showed that deletion of *C*‐mannosylation in the N‐terminal TSR1 domain (ADAMTS1/2WF) dramatically decreases enzymatic activity. The loss of *C*‐mannosylation might alter the folding of the protein, resulting in weaker substrate recognition.

VM formation was significantly increased in NEC8‐ADAMTS1‐MH cells compared with that of parent cells but not in NEC8‐ADAMTS1/2WF cells (Fig. 4A,B). It could be explained by the following model: Although ADAMTS1 has a function that turns LAP‐TGF‐β into the activated form, as shown in Fig. 3C, secretion is decreased in ADAMTS1/2WF. Therefore, it is possible that the amount of cleaved LAP‐TGF‐β is reduced in ADAMTS1/2WF compared to the wild‐type. Activated TGF‐β induces epithelial‐mesenchymal transition (EMT) [42]; thus, the overexpression of wild‐type ADAMTS1 resulted in progressive EMT, while ADAMTS1/2WF did not. Since EMT is thought to positively regulate VM formation [43], VM formation ability was increased in NEC8‐ADAMTS1‐MH cells compared to the parent but not in NEC8‐ADAMTS1/2WF‐MH cells. As shown in Fig. 4C, the accumulation of pro‐ADAMTS1/2WF did not interfere with intracellular signaling pathways known to contribute to VM formation, suggesting that the active form of ADAMTS1 is responsible for promoting VM. Therefore, further investigation into the relationship between ADAMTS1 and VM is warranted. Considering the fact that *C*‐mannosylation of ADAMTS1 affected its secretion, ECM degradation activity, and VM formation, this study suggests that *C*‐mannosylation in ADAMTS1 might be a promising target for human testis germ tumor therapy.

## Conflict of interest

The authors declare no conflict of interest.

## Author contributions

TK, RK, and NH conceived and performed the experiments, analyzed the data, and wrote the manuscript. TS and ND performed the LC–MS/MS analysis. RK, ND, and SS conceived and designed the experiments and wrote the manuscript. All authors have discussed the results, commented on the manuscript, and have read and approved the final manuscript.

## Supporting information


**Fig. S1.** Structural comparison of wild‐type and mutated ADAMTS1.

## Data Availability

The data used and analyzed during the current study are available from the corresponding author upon reasonable request.
