# Supplementary material for: C‐mannosylation promotes ADAMTS1 activation and secretion in human testicular germ cell tumor NEC8 cells
Source: FEBS Lett. 2025 Aug 6;599(21):3113–23. doi: 10.1002/1873-3468.70133 (PMC12599623; doi:10.1002/1873-3468.70133)
Supplement: Supplementary file 1 — Fig. S1. Structural comparison of wild‐type and mutated ADAMTS1. [file FEB2-599-3113-s001.docx]

Fig. S1. Structural comparison of wild-type and mutated ADAMTS1.

(A,B) Conformational status of ADAMTS1-wild-type (WT) (A) and ADAMTS1-W562F,W565F (2WF) by AlphaFold 3 (default settings) were represented. The left panel shows the overall structure, and the right panel presents a magnified view of the amino acid site undergoing *C*-mannosylation.
